# Refining risk prediction in pediatric acute lymphoblastic leukemia through DNA methylation profiling

**DOI:** 10.1186/s13148-024-01662-6

**Published:** 2024-03-28

**Authors:** Adrián Mosquera Orgueira, Olga Krali, Carlos Pérez Míguez, Andrés Peleteiro Raíndo, José Ángel Díaz Arias, Marta Sonia González Pérez, Manuel Mateo Pérez Encinas, Manuel Fernández Sanmartín, Daniel Sinnet, Mats Heyman, Gudmar Lönnerholm, Ulrika Norén-Nyström, Kjeld Schmiegelow, Jessica Nordlund

**Affiliations:** 1 Department of Hematology, University Hospital of Santiago de Compostela, Compostela, Spain; 2 Health Research Institute of Santiago de Compostela, Compostela, Spain; 3 Department of Medical Sciences, Molecular Precision Medicine, Uppsala University, Uppsala, Sweden; 4 Science for Life Laboratory, Uppsala University, Uppsala, Sweden; 5 Department of Pediatric Medicine, University Hospital of Santiago de Compostela, Santiago de Compostela, Spain; 6 Research Center, CHU Sainte-Justine, Montréal, Canada; 7 Department of Pediatrics, Université de Montréal, Montreal, Canada; 8 Childhood Cancer Research Unit, Karolinska Institutet, Astrid Lindgren Children’s Hospital, Karolinska University Hospital, Stockholm, Sweden; 9 Department of Women’s and Children’s Health, Uppsala University, Uppsala, Sweden; 10 Department of Clinical Sciences, Pediatrics, Umeå University, Umeå, Sweden; 11 Pediatrics and Adolescent Medicine, Rigshospitalet, and the Medical Faculty, Institute of Clinical Medicine, University of Copenhagen, Copenhagen, Denmark; 12 For the Nordic Society of Pediatric Hematology and Oncology (NOPHO), Stockholm, Sweden

**Keywords:** Pediatric acute lymphoblastic leukemia, Epigenetics, DNA methylation, Machine learning, Artificial intelligence, Relapse risk, Mortality risk, Precision medicine

## Abstract

Acute lymphoblastic leukemia (ALL) is the most prevalent cancer in children, and despite considerable progress in treatment outcomes, relapses still pose significant risks of mortality and long-term complications. To address this challenge, we employed a supervised machine learning technique, specifically random survival forests, to predict the risk of relapse and mortality using array-based DNA methylation data from a cohort of 763 pediatric ALL patients treated in Nordic countries. The relapse risk predictor (RRP) was constructed based on 16 CpG sites, demonstrating c-indexes of 0.667 and 0.677 in the training and test sets, respectively. The mortality risk predictor (MRP), comprising 53 CpG sites, exhibited c-indexes of 0.751 and 0.754 in the training and test sets, respectively. To validate the prognostic value of the predictors, we further analyzed two independent cohorts of Canadian (*n* = 42) and Nordic (*n* = 384) ALL patients. The external validation confirmed our findings, with the RRP achieving a c-index of 0.667 in the Canadian cohort, and the RRP and MRP achieving c-indexes of 0.529 and 0.621, respectively, in an independent Nordic cohort. The precision of the RRP and MRP models improved when incorporating traditional risk group data, underscoring the potential for synergistic integration of clinical prognostic factors. The MRP model also enabled the definition of a risk group with high rates of relapse and mortality. Our results demonstrate the potential of DNA methylation as a prognostic factor and a tool to refine risk stratification in pediatric ALL. This may lead to personalized treatment strategies based on epigenetic profiling.

**Supplementary Information:**

The online version contains supplementary material available at 10.1186/s13148-024-01662-6.

## Introduction

Acute lymphoblastic leukemia (ALL) is the most common childhood malignancy, representing 25% of all cancers. ALL exhibits clinical and biological heterogeneity, driven by recurrent genetic aberrations [1]. Treatment advancements have led to 5-year overall survival (OS) rates exceeding 90% [2]. However, relapsed patients face slower progress, with an mortality rate of approximately 45% in Nordic countries [3]. Additionally, ALL treatments carry risks of adverse outcomes, including increased late incidence of secondary malignancies, as well as long-term neurological, cardiac, endocrine, and social/psychological disorders [4]. In this regard, the long-term organic complications associated with an allogeneic stem cell transplantation (allo-HCT) during childhood are broad [5], and therefore optimizing patient selection is key to minimize unwanted toxicity.

Upfront treatment is primarily based on combination chemotherapy. Prognostic factors have been used to estimate the risk of relapse and to adjust treatment intensity accordingly, which has resulted in reduced toxicity without adversely impacting the rate of curation [6]. As the treatment intensity required for cure varies greatly between patients, a risk-adapted strategy is intended to reduce toxicity for those cases that are likely to achieve curation with low-dose chemotherapeutics, while more intense schemes are reserved for high-risk groups [7–15]. Prognostic factors for risk stratification include age, white blood cell (WBC) count, immunophenotype, minimal residual disease (MRD), cytogenetic aberrations, and central nervous system (CNS) involvement [2, 7, 9, 16]. Additional factors, like *IKZF1* deletion, may enhance future risk prediction [17, 18]. Intensive chemotherapy and cell therapy (allo-HCT and chimeric antigen receptor (CAR)-T cell) are used for relapsed and refractory disease [19–21].

Genomic techniques have the potential to improve risk stratification [22, 23], as traditional risk grouping approaches may not be applicable to all circumstances [24–26]. High-dimensional data cluster patients and assess their relationship with drug response and survival [27, 28]. However, the complex molecular determinants of leukemia hinder accurate grouping, resulting in misclassification. An optimization problem simplifies the analysis by incorporating clinical outcomes and baseline prognostic information to derive a risk predictor for predicting outcomes in new patients [29]. This approach has paved the way for the development of prognostic and predictive tools in various onco-hematological fields, including myelofibrosis, myelodysplastic neoplasms, and multiple myeloma [30–32].

While genetic changes have led to a better understanding of tumor biology, epigenetics has emerged as a valuable avenue to explain tumor phenotypes. The epigenetic landscape is essential in defining tumor types and subtypes, allowing for high-resolution classification and insight into tumor-specific mechanisms [33]. In B-cell malignancies, epigenetic alterations involve distinct cellular processes and B-cell-specific aspects, enabling accurate detection of B-cell tumor-specific aberrations for improved prognostication [26, 34, 35]. ALL cells are known to exhibit CpG island hypermethylation [36], but minimal global loss of methylation, a fact which was particularly marked in T-cell ALL [37]. While the mechanisms underlying the transformation of progenitor B- and T-cells into leukemic cells are not fully understood, these studies cumulatively demonstrate the potential of DNA methylation as a biomarker for lineage and subtype classification, prognostication, and disease progression [38].

In the present study, we trained two machine learning (ML) models based on DNA methylation signatures obtained at ALL diagnosis aimed to refine risk grouping. Our results suggest that DNA methylation profiling at ALL diagnosis could aid in future refinement of risk assignment and may contribute to improved survival and long-term quality of life for pediatric ALL patients.

## Materials & methods

### Data origin and preprocessing

Pediatric ALL samples from three cohorts were processed as originally described by Nordlund et al. (2013) [26], Busche et al. [39] and Krali et al. [40] The *Nordlund *et al. dataset was used to build the risk predictors and evaluate their performance internally. This dataset comprises of pre-treatment DNA methylation status of a filtered set of 435,941 CpG sites downloaded from GEO (GSE49031), which assayed 763 diagnostic ALL samples on Infinium Human Methylation 450K BeadChips (450k array) [26]. The following clinical covariates were available: age, sex, Down syndrome status, risk group and cytogenetic subtype. The risk group was defined according to age at diagnosis, WBC count, B- or T-lineage, and genetic aberrations according to the NOPHO-92 or NOPHO-2000 protocols [6, 16]. Patients were assigned to standard, intermediate or high-risk groups and treated accordingly. Relapse free survival (RFS) was established from the time of ALL diagnosis to the date of the first relapse. OS was defined as the time from ALL diagnosis to the moment of death from any cause.

For external validation, we identified and downloaded the following ALL datasets generated on the 450k array: *Busche *et al*.* GSE38235 (*n* = 42) [39], and *Krali *et al*.* (10.17044/scilifelab.22303531) (*n* = 384) [40]. RFS and OS were defined as indicated above also for the external validation datasets. The dataset by *Busche *et al*.* included complete 450k array and clinical data from 42 Canadian BCP-ALL patients treated between 1999 and 2010 at the Sainte-Justine University Health Center (UHC; Montreal, QC, Canada). All patients underwent treatment with uniform Dana-Farber Cancer Institute ALL Consortium protocols DFCI 95–01, 2000–01 or 2005 [41–43]. The cohort by *Krali *et al*.* included patients treated with the NOPHO-2000 and NOPHO-2008 protocols [12, 40].

### Variable selection and model development

**Fig. 1:**
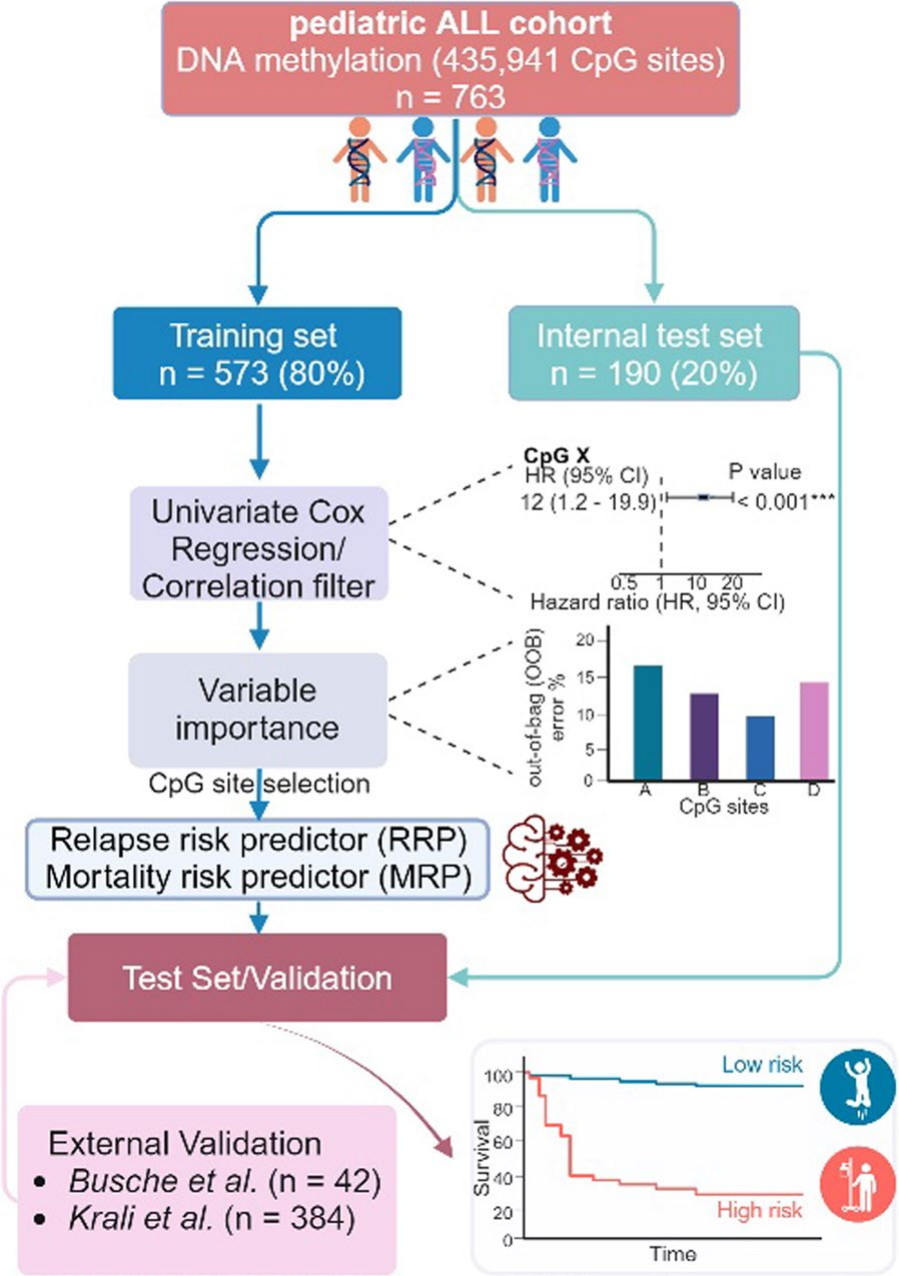
Graphical representation of the study design. The models were trained with data from 763 ALL patients, all of whom had previously been characterized by genome-wide DNA methylation arrays. The dataset was partitioned into a training set (80% of the patients) and a test set (the remaining 20%). The training set was used to identify CpG sites with DNA methylation status associated with two key outcomes: relapse risk and mortality. The selected CpG sites were used to train Random Survival Forests models. Two models were generated: a Relapse Risk Predictor (RRP) and a Mortality Risk Predictor (MRP). The test set was utilized for internal validation. Finally, the models were further validated on two additional datasets

The cohort by *Nordlund *et al*.* [26] was randomly divided into a training (80% of the cohort, *n* = 573) and a test (20% of the cohort, *n* = 190) sets. Univariate Cox regression (survival package) [41] was used to evaluate the association of CpG sites with RFS and OS in the training set. CpGs were selected according to the role of a filter based on a hazard ratio (HR) < 0.1 or > 10 for CpG site selection. CpG selection was based on the univariate association (cox regression) of the DNA methylation beta-values of each CpG site with RFS and OS in the training set. CpGs with *q*-values < 0.01 or 0.05 were selected for model construction with or without a HR filter. Due to collinearity, a correlation filter was applied to the mortality risk predictor (MRP), in contrast to the relapse risk predictor (RRP), which did not require correlation filtering to reduce dimensionality due to its smaller size. This filter removed CpGs with a Pearson’s correlation > 0.7 with any other variable included in the regression. Multivariate models of survival were constructed using random forests (randomForestSRC package) [44]. The model outputs include a survival function and a cumulative hazard function, which represent patient risk predictions over time. Missing variables were imputed in each dataset separately using a missing data algorithm developed by *Ishawarian *et al. [42] Random forests were created with 1,000 trees. Hyperparameter optimization of the *mtry* and *nnodes* variables was performed using a grid search method. Variable importance was calculated with the permutation importance method (also known as Breiman-Cutler method, implemented in the *vimp* function) and used to eliminate those CpGs with lower predictive value. In random forests, variable importance is commonly evaluated using a permutation-based method. Initially, the model's out-of-bag (OOB) error is calculated. For each feature, its values are then randomly shuffled in the OOB dataset, and a new OOB error is computed using the perturbed data. The difference between the new and original OOB errors gives the VIMP score for that feature, with higher scores indicating greater importance. A graphical summary of the workflow is represented in Fig. 1.

The discriminative capacity of the random forest models in the training set was evaluated with OOB error estimates of the concordance index (c-index) and with time-dependent areas under the receiving operator (ROC) curve (AUCs). The c-index is a metric for survival prediction and reflects a measure of how well a model predicts the ordering of patients’ event times (e.g., death or relapse). A c-index of 0.5 represents a random model, whereas a c-index of 1 refers to a perfect ranking between real and predicted outcomes. OOB is based on subsampling with replacement to create training samples for the model to learn from. OOB error is the average prediction error on each training sample *Xi*, using only the trees that did not have *Xi* in their bootstrap sample. For OOB error estimations, sampling was performed without replacement, which by default takes 0.632 times the sample size. Time-dependent AUCs were calculated using cross-validated cox regression with predicted cumulative hazards as independent variables [45]. Cross-validation was performed with the *bootcv* algorithm using 500 cycles. In each cycle, 75% of samples were used for training and 25% for testing. In the particular case of the training set, all random forest predictions used as input for downstream analysis were OOB to avoid overfitting of the risk predictions during the training phase of the model. For calibration, continuous rank probability scores (CRPS) were calculated as the integrated Brier score divided by time.

The *surv_cutpoint* function (survminer package) [46] was used to identify the optimal cut-off for the MRP/RRP scores. This approach is centered on outcomes and aims to identify a cut point value that exhibits the highest level of statistical significance concerning its association with the variable of outcome, specifically in the context of survival analysis.

## Results

### Cohort description and variable selection process

**Fig. 2:**
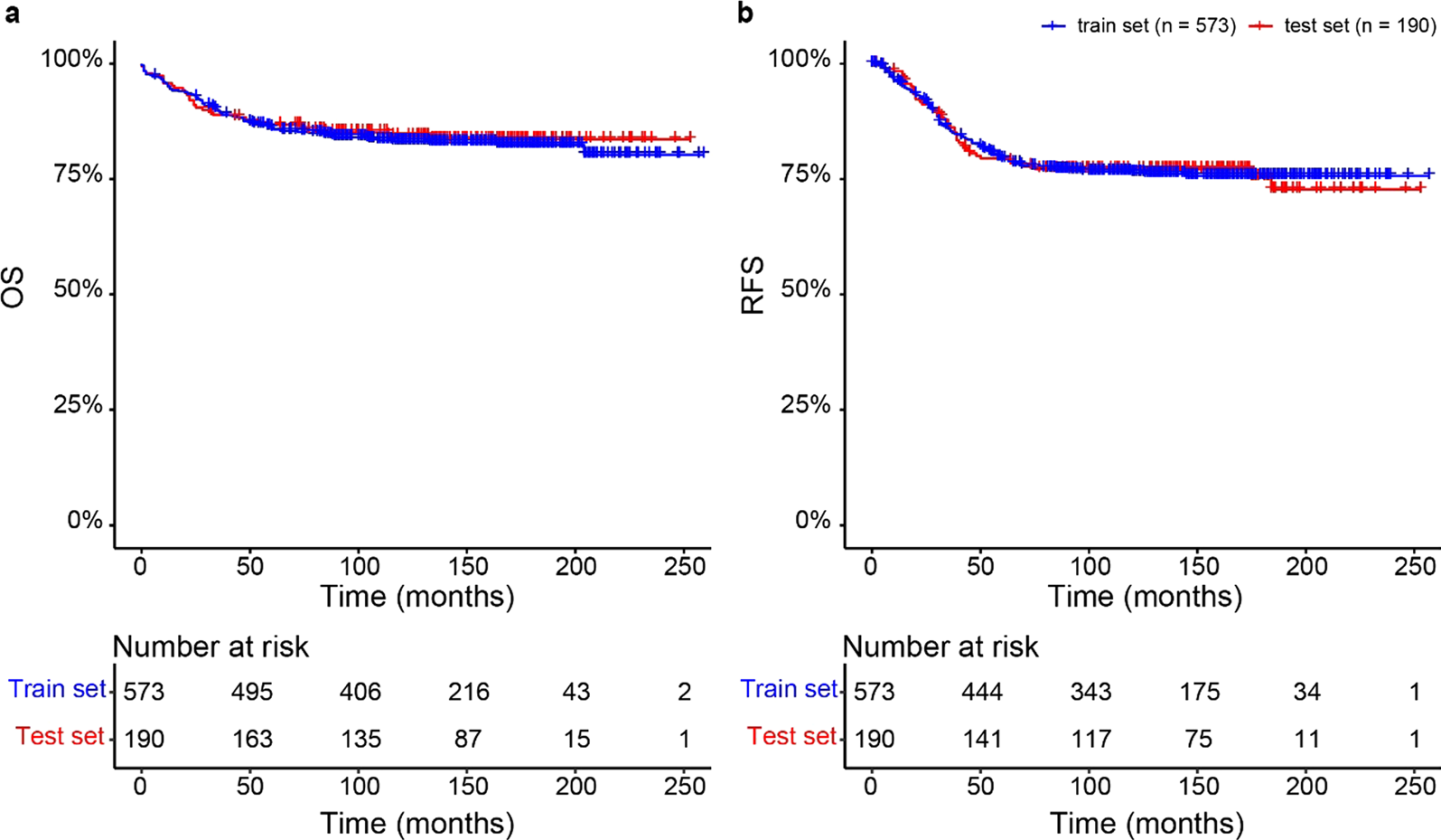
Kaplan–Meier survival analysis representing overall survival and relapse-free survival in the training and test sets. **a** Kaplan–Meier estimates for overall survival (OS) for the training (blue) and test (red) datasets. **b** Kaplan–Meier estimates for relapse free survival (RFS) for the training (blue) and test (red) datasets

Patient characteristics, including cytogenetic subtype classifications and clinical information for the training and test sets are presented in Table 1 and Additional file 2: Table S1. Kaplan–Meier curves for OS and RFS for each of the training and test cohorts with data available are shown in Fig. 2. Random survival forests for death and relapse risk prediction were constructed, and ordered according to their c-indexes (Additional file 2: Table S2). The RRP with best performance was based on 29 CpGs with a *q*-value threshold < 0.05 and the MRP with best performance was based on 174 CpGs with *q*-values < 0.05, combined with a correlation filter (Additional file 2 Table S2).

**Table 1:** Baseline characteristics of the patients

	Training set	Test set
*N*	573	190
Median follow-up	145 months	159 months
% of Allo-HCT	55 (9.6%)	10 (5.3%)
Relapses (%)	129 (22.5%)	43 (22.6%)
Deaths (%)	97 (16.9%)	30 (15.8%)
Median age (range)	5 (0–19)	5 (0–18)
Sex (Male/Female)	329 (57.4%)/244 (42.6%)	103 (54.2%)/87 (45.8%)
Down syndrome	17 (2.97%)	2 (1.05%)
Standard Risk	157 (27.4%)	50 (26.3%)
Intermediate Risk	196 (34.2%)	70 (36.8%)
High Risk	206 (35.9%)	64 (33.7%)
Infant	15 (2.4%)	5 (2.6%)

### Relapse risk prediction using random forests

The training and test sets contained 129 and 43 relapse events each. In the training set, 29 CpGs were univariately associated with RFS (*q*-value < 0.05, Additional file 2: Table S2). Using additional dimensionality reduction techniques based on variable importance for random forests, we reduced the signature to 16 CpG dinucleotides (Table 2, Additional file 2: Table S3). The RRP achieved c-indexes of 0.667 and 0.677 in the training and test sets, respectively (Additional file 1: Figs. S1 and S2). When adding the cytogenetic subtype (c-indexes, 0.667 and 0.683 for the training and test sets, respectively) or age at diagnosis (c-indexes, 0.674 and 0.672) as covariates, the model performance did not change considerably. Overall, the prognostic impact of cytogenetic classification alone across the entire cohort was low for RFS (c-index, 0.512). The RRP model was also predictive in T-cell ALL (c-indexes, 0.699 and 0.603 in the training (*N* = 77) and test (*N* = 24) sets).

**Table 2:** Annotation of the 450K-panel probes included in the relapse risk predictor (RRP)

CpG ID	Gene symbol	Location	CpG annotation
cg20324356	CARS1	11p15.4	N_Shore
cg23672291	GTF2F2	13q14.12	CpG island
cg16267059	MFAP1	15q15.3	Transcription Start Site
cg00046913	HAGH	16p13.3	Transcription Start Site
cg18076500	ECSIT	19q13.2	Transcription Start Site
cg08445782	ZNF217	20q13.2	N_Shelf
cg14396214	ZGPAT	20q13.33	1st Exon
cg08025954	SPECC1L-ADORA2A	22q11.23	Transcription Start Site
cg17209692	KLHL29	2p24.1	CpG Island
cg04956471	DGKD	2q37.1	Gene Body
cg18358754	GEMIN5	5q33.2	CpG island
cg25663770	NCR2	6p21.1	Transcription Start Site
cg03446203	SNHG32 / HSPA1B	6p21.33	Transcription Start Site
cg22535729	RP1	8q12.1	CpG island
cg10286363	RPS20	8q12.1	CpG island
cg21173721	FAM102A	9q33.11	Transcription Start Site

### Longitudinal assessment of the relapse risk predictor

**Fig. 3:**
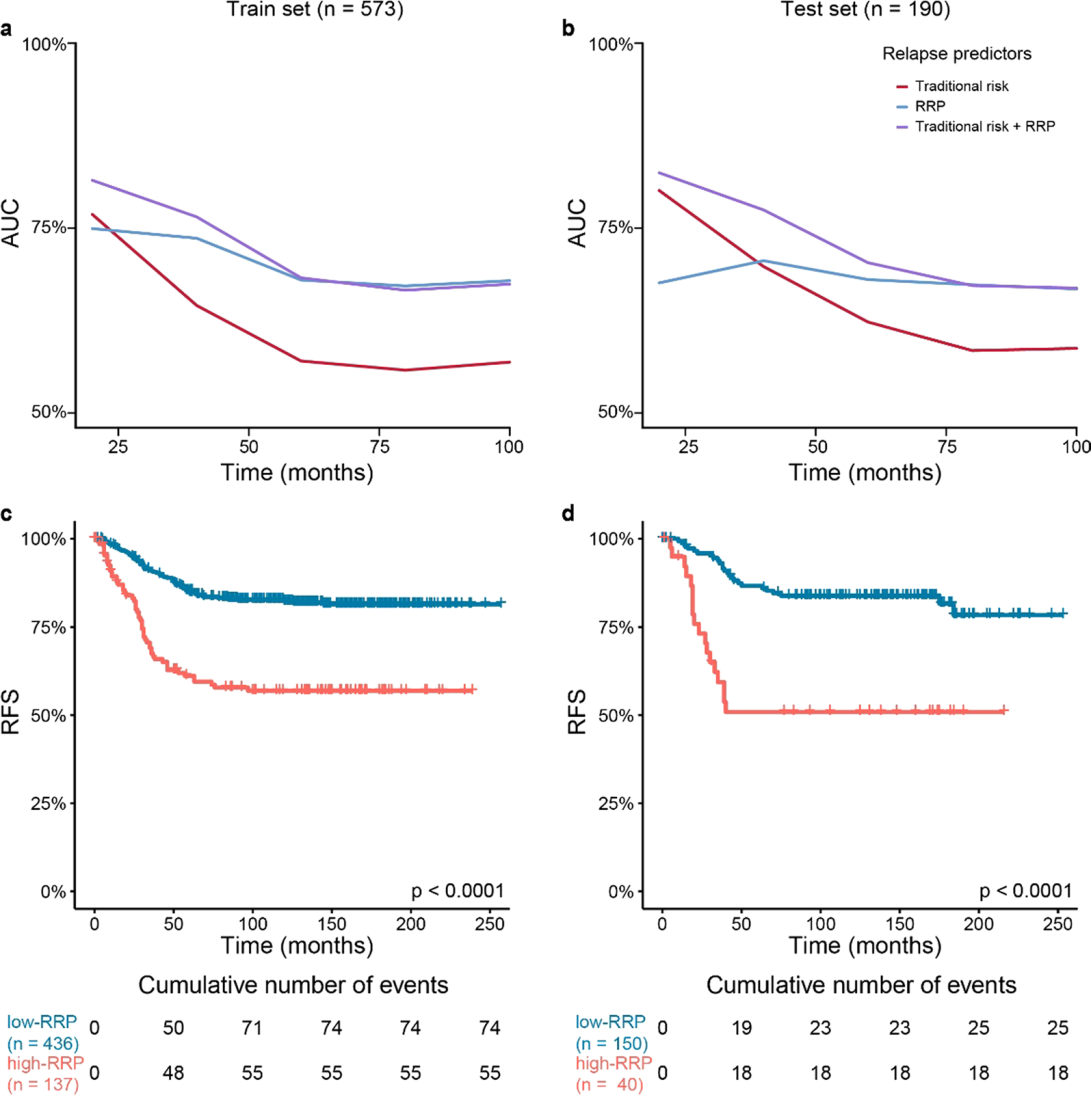
Time-Dependent Area Under the Curve and Kaplan-Meier Plots for Relapse-Free Survival Analysis. **a–b** Time-dependent Area Under the Curve (AUC) representing the accuracy of the cox models in the prediction of relapse free survival (RFS) in the training (a) and test (b) sets. The red line represents a cox  model based on standard of care risk grouping, the blue line represents the cox model based on the relapse risk predictor (RRP), and the purple line represents the cox model integrating both methods. **c–d** Kaplan-Meier plots depicting the RFS for the patients assigned to the two groups denoted by the relapse risk predictor: high-RRP (coral line) and low-RRP (blue line) in the training (c) and test (d) sets

The scores of the RRP were implemented on cross-validated cox models for the calculation of time-dependent AUCs. We compared the model to clinical risk groups, and the results of the former outperformed the latter in terms of AUCs and bootstrapped c-indexes in the training and test sets (Fig. 3a–b). The clinical risk grouping more accurately predicted early relapses, but with a drop in performance after 20 months. The RRP, however, remained superior and more stable even after 20 months. The combination of the RRP with clinical risk grouping provided the best prognostic accuracy, outperforming any of the two separate strategies. Remarkably, the 20-month AUC was 81.4% and 82.5% in the training and test sets. To improve interpretability and enhance applicability, we identified the optimal cut-point of the RRP score in the training set (14.09 points). This was performed in order to divide patients into high- and low-RRP groups. The same cut-off (14.09) was also applied on the test set. The high-RRP group demonstrated a significant difference in relapse rates in comparison with the low-RRP (*p*-value < 0.001, Fig. 3c–d, Additional file 2: Table S4).

### Mortality risk prediction using random forests

The training and test sets contained 97 and 30 deaths, respectively. In the training set, 174 CpGs were univariately associated with OS (*q*-value < 0.05) accompanied by HRs < 0.1 or > 10. After collinearity filtering, the MRP signature consisted of 53 CpG sites (Table 3, Additional file 2: Table S5). The MRP achieved c-indexes of 0.751 and 0.754 in the training and test sets, respectively (Additional file 1: Figs. S3 and S4). Similarly to the RRP, addition of cytogenetic subtype (c-indexes, 0.753 and 0.751 for the training and test sets, respectively) or age at diagnosis (c-indexes, 0.752 and 0.753) as a covariate did not alter the performance of the MRP. However, the prognostic impact of cytogenetic classification alone in the entire cohort was low for OS (c-index, 0.597). Furthermore, we again observed that the MRP was also prognostic in the subgroup of patients with T-ALL (c-indexes, 0.702 and 0.597 in the training and test sets). Finally, we observed that the MRP signature could also be used to predict relapse (c-index of 0.694 and 0.643 in the training and test sets, respectively), but the RRP could not be used to predict OS.

**Table 3:** Annotation of the 450K-probes probes included in the mortality risk predictor (MRP)

CpG ID	Gene Symbol	Location	CpG Annotation
cg13901752	OPTN / CCDC3	10p13	Transcrition Start Site
cg27210565	SEPT7P9	10q11.1	Transcrition Start Site
cg04444771	RASSF4 / DEPP1	10q11.21	Gene Body
cg27139956	RASSF4	10q11.21	CpG Island
cg07260003	VSTM4	10q11.23	Gene Body
cg20534287	ECHS1 / PAOX	10q26.3	Transcrition Start Site
cg24496614	BEST1 / FTH1	11q12.3	Transcrition Start Site
cg03033176	EHBP1L1	11q13.1	S_Shore
cg16110032	Intergenic	11q13.5	N_Shelf
cg14199423	CHD4 / LPAR5	12p13.31	Unclassified
cg14541870	SETD1B	12q24.31	S_Shore
cg23743428	MIPEPP3	13q12.11	CpG Island
cg01911068	PCDH9	13q21.32	Transcrition Start Site
cg01363662	ODF3L1	15q24.2	Transcrition Start Site
cg09363128	GSE1	16q24.1	Unclassified (cell-tye specific)
cg08374494	ZFPM1	16q24.2	N_Shelf
cg23430664	XYLT2	17q21.33	CpG Island
cg12296532	ACTG1	17q25.3	CpG Island
cg00671759	ACTG1	17q25.3	CpG Island
cg04384209	KDM4B	19p13.3	N_Shelf
cg19726840	DAZAP1 / RPS15	19p13.3	Transcrition Start Site
cg19599529	GMFG	19q13.2	3'UTR
cg26587014	BCL3	19q13.32	S_Shelf
cg15679331	PRTM1 / BCL2L12	19q13.33	Transcrition Start Site
cg19675684	AKIRIN1	1p34.3	Transcrition Start Site
cg08262220	PRDM16	1p36.32	N_Shore
cg11096441	ADNP / DPM1	20q13.13	Transcrition Start Site
cg06454380	c22orf34	22q13.3	S_Shelf
cg14911521	VAX2 / LINC01143	2p13.3	N_Shore
cg10633958	SLC8A1	2p22.1	N_Shore
cg00866476	MAL / MRPS5	2q11.1	N_Shore
cg00992239	MME	3q25.2	Transcrition Start Site
cg27177997	RBPJ	4p15.2	S_Shore
cg24877510	CNOT6L	4q21.1	S_Shore
cg02763617	CWC27	5q12.3	N_Shore
cg25149751	PPP2R2B	5q32	Transcrition Start Site
cg01993576	SLC29A1	6p21.1	S_Shore
cg16323034	Intergenic	6p21.2	Unclassified
cg08576623	BRPF3	6p21.31	S_Shore
cg24536691	VPS52	6p21.32	Transcrition Start Site
cg03891050	LSM2	6p21.33	S_Shore
cg17718302	H3C12 / H2AC17 / H2BC17	6p22.1	Transcrition Start Site
cg02959285	HIST1H2BG	6p22.2	Transcrition Start Site
cg07090714	Intergenic	6p25.1	Unclassified (cell-tye specific)
cg23448978	COBL	7p12.1	Gene Body
cg27377289	UMAD1	7p21.3	Unclassified (cell-tye specific)
cg09926212	ORAI2	7q22.1	Transcrition Start Site
cg02514021	LINC-PINT	7q32.3	CpG Island
cg04301738	LINC00996	7q36.1	Unclassified
cg08261702	RP11-511P7.5	7q36.1	N_Shore
cg13072214	PARP10	8q24.2	Transcrition Start Site
cg00470505	DIPK1B / AGPAT2	9q34.3	CpG Island
cg03180426	SAPCD2	9q34.3	CpG Island

### Longitudinal assessment of the mortality risk predictor

**Fig. 4:**
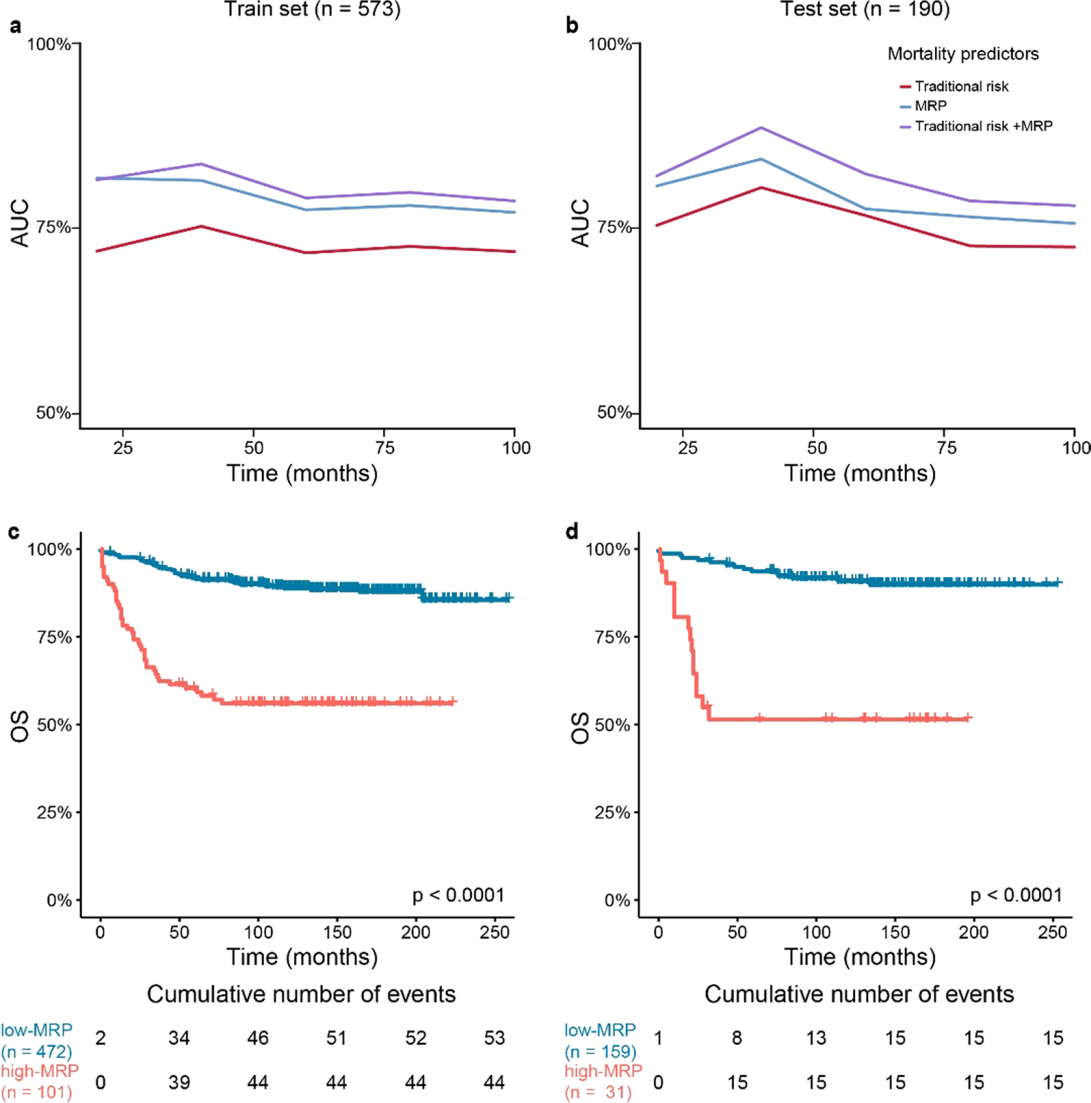
Time-Dependent AUCs and OS Kaplan-Meier Plots for Overall Survival Prediction. **a-b** Time-dependent AUCs representing the accuracy of the different classifiers (cox regression) in the prediction of OS for the training **a** and test **b** sets. The red line represents the cox model based on standard risk groups, the blue line represents the cox model based on the mortality risk predictor (MRP) and the purple line represents the cox model integrating both methods. **c-d** OS Kaplan-Meier plots for the high-MRP (coral line) and the low-MRP (blue line) groups, as determined by the surv_cutpoint MRP optimal cut-off, in the training **c** and test **d** sets

The scores generated by the MRP were implemented on cross-validated cox models for the calculation of time-dependent AUCs. We compared this model with the conventional clinical risk grouping. Once again, the results of the MRP outperformed the clinical risk grouping strategy in terms of AUCs and bootstrapped c-indexes in both patient groups. In this case, the MRP outperformed the conventional risk grouping strategy at all evaluated time points (Fig. 4a–b). The combination with clinical risk grouping provided the best prognostic accuracy, outperforming any of the two individual strategies. The highest accuracy of the MRP was observed for risk stratification at 40 months post-diagnosis, which rendered AUCs of 83.66% and 88.58% in the training and test sets. We calculated the optimal MRP cut-off (12.31 points) to split the patients into low- and high-MRP groups. The high-MRP group had significantly shorter OS than the low-MRP group in both the train and test datasets (Fig. 4c–d, Additional file 2: Table S4).

### Validation

We identified two independent datasets of 450k array data generated from pediatric ALL cohorts, which we used to validate the predictors. In the *Busche *et al*.* data set (*n* = 42) five patients relapsed during follow-up, and two deaths were recorded. Due to the small number of deaths, we used this dataset to validate only the prediction performance of the RRP, achieving a c-index 0.667. In the *Krali *et al*.* dataset (*n* = 384, Additional file 1: Fig. S5, Additional file 2: Table S6) 50 patients relapsed during follow-up and 45 patients died, of which 19 did so due to relapse and 20 were in complete remission. The RRP and MRP were 0.529 and 0.621, respectively for the *Krali *et al*.* dataset. In this dataset, the RRP score was weakly associated with relapse risk (*p*-value 0.064, HR 1.028 (95% CI: 0.9984–1.058 for each risk unit increase), while the MRP score was strongly associated with OS (*p*-value 1.04 × 10^–4^, HR 1.073 (95% CI: 1.036–1.112 for each risk unit increase). We observed that the MRP provided its best prognostic accuracy within the standard and infant risk groups (Additional file 2: Table S7). Furthermore, when we applied the MRP on the RFS data, the c-index for predicting risk of relapse in the validation data (0.62 for *Krali *et al*.*) was similar to the cross-prediction performance observed in the train and test sets.

**Fig. 5:**
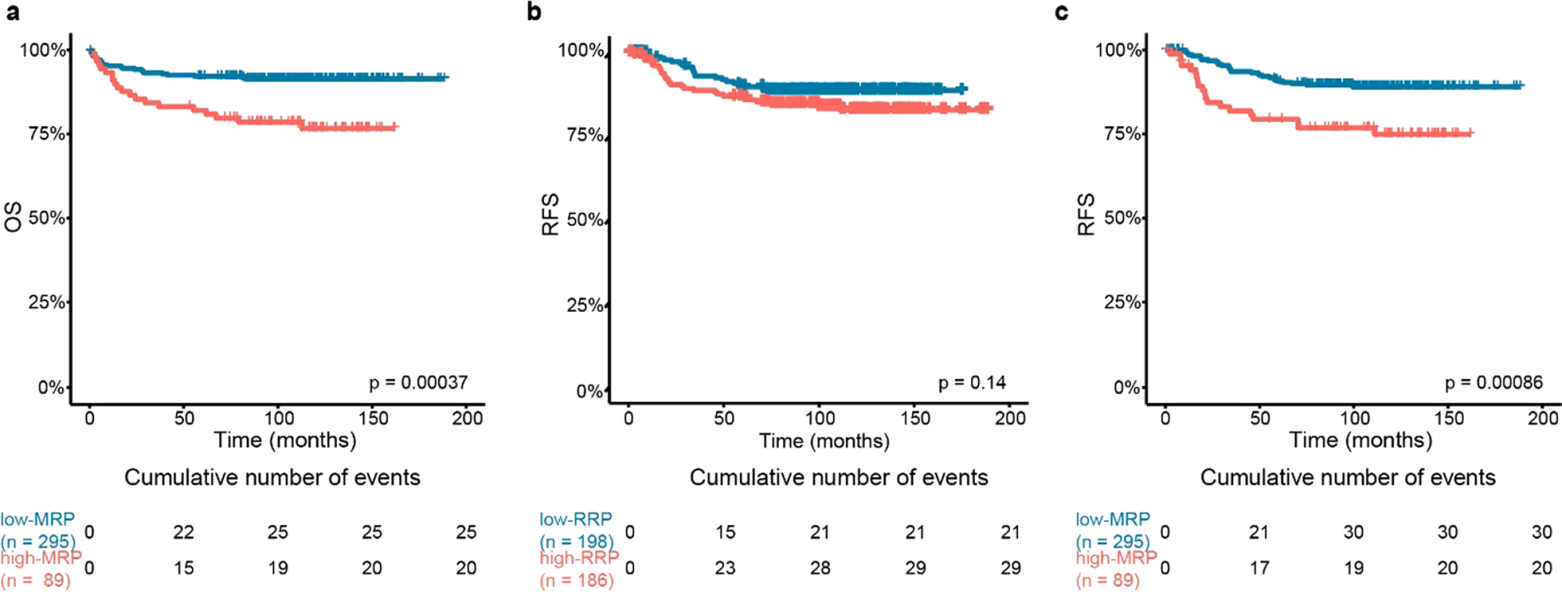
Kaplan-Meier plots for overall and relapse-free survival in the independent dataset. **a–c** Kaplan-Meier plots for **a** overall survival (OS) and **b-c** relapse-free survival (RFS) in the independent dataset **a** OS differences between the high-MRP; (coral line) and low-MRP (blue line) groups. **b** RFS differences between patients assigned by the model to the high-RRP, (coral line) and low-RRP, (blue line) groups. **c** RFS differences between patients assigned to the high-MRP (coral line) and low-MRP (blue line) groups

We applied the previously defined low- and high-MRP dichotomization to evaluate differences in OS within this cohort. Consistently, increased mortality was observed in the high-MRP group (*p*-value < 0.001, Fig. 5a, Additional file 2: Table S4). Along the same line, we employed the low- and high-RRP dichotomization, but no significant difference in RFS was observed between the groups (*p*-value 0.14, Fig. 5b, Additional file 2: Table S4). Hence, we used the MRP groups to investigate if the MRP dichotomization could be predictive of relapse, which resulted in significant differences in RFS between the low-MRP and low-RRP group (*p*-value < 0.001; Fig. 5c, Additional file 2: Table S4).

### Patient characteristics associated with epigenetic risk

**Fig. 6:**
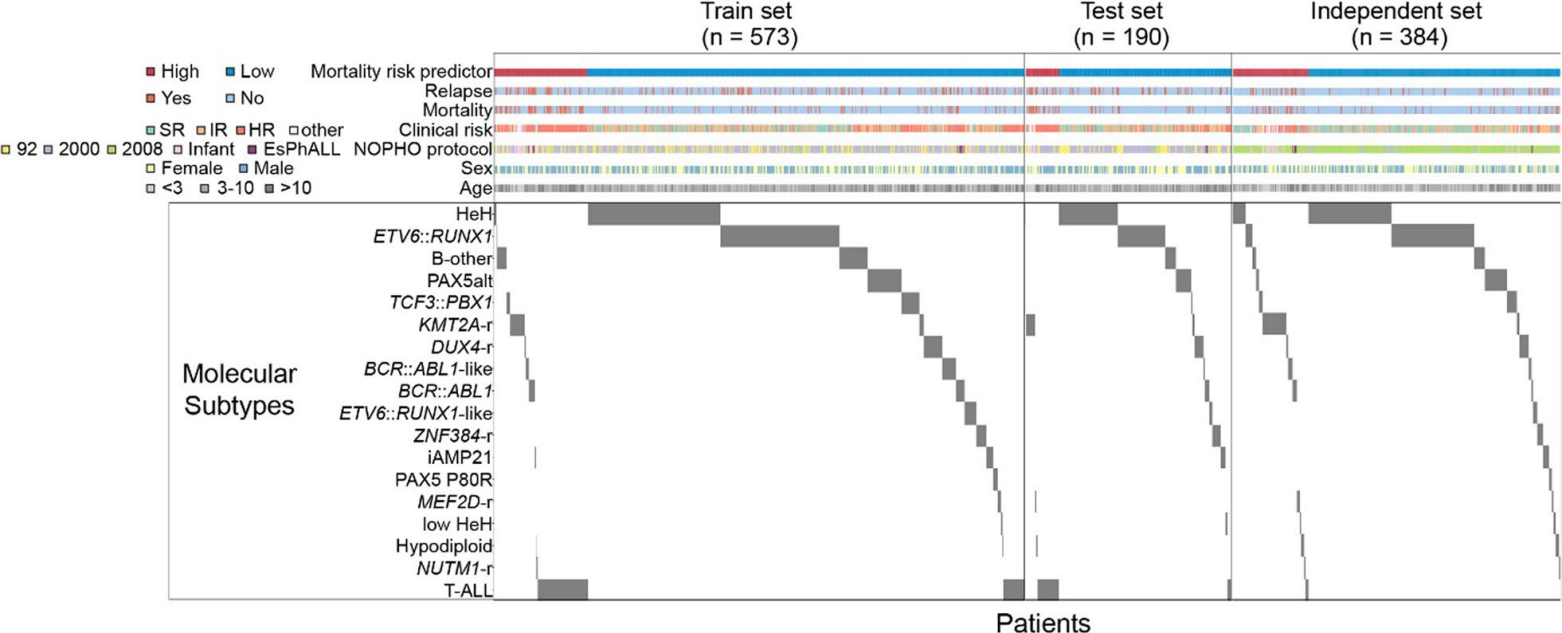
Clinical outcome and molecular subtypes of patients in the train (left, *n* = 573), test (center, *n* = 190) and independent datasets (right, *n* = 384). The patients were sorted by the mortality risk predictor (MRP) groups (high/low, *x*-axis). Clinical annotations, including relapse, mortality, clinical risk groups, NOPHO treatment protocol, sex and age are provided as annotation bars, color-coded according to the figure legend. Patient molecular subtypes (*y*-axis) are denoted as gray vertical lines on the heatmap plots

Based on the current ICC system for ALL subtyping [47] we grouped the 1,147 ALL patients from the training, test, and validation sets using the latest molecular classification. The frequencies of the cytogenetic profiles were consistent with those described across other ALL cohorts [1, 48–51]. Using the dichotomized MRP cut-point, each sample set visualized by the MRP grouping (Fig. 6). Patients in the high-MRP group had a tendency to display to the known high risk molecular subtypes (T-ALL, *BCR*::*ABL1*, *KMT2A*-r, hypodiploid, and *MEF2D*-r), while low-risk molecular subtypes (HeH, *ETV6*::*RUNX1*, and PAX5-alteration) were more frequent in the low-MRP group. Patients denoted as B-other were split between the high-MRP and low-MRP groups. Notably, in the independent cohort, patients characterized by standard-risk cytogenetic aberrations, such as high hyperploid and *ETV6*::*RUNX1*, were assigned to the high-MRP group.

## Discussion

This study provides evidence that DNA methylation signatures analyzed using ML algorithms offer a promising avenue for personalized risk stratification in pediatric ALL patients. Our model effectively predicted patient risk using a small set of CpG sites, demonstrating an improvement over conventional prognostic approaches. Integration of the ML predictors with the conventional clinical risk score resulted in enhanced overall performance. Notably, the mortality risk predictor (MRP) outperformed the relapse risk predictor (RRP), potentially indicating the superior predictive ability of DNA methylation patterns in assessing biological risk. Importantly, for patients initially treated with low or standard-risk protocols who later relapsed and received intensified therapy, a substantial fraction achieved complete response. Hence, risk-associated DNA methylation signatures could help identify highly refractory patients who are unlikely to respond adequately to salvage chemotherapeutics.

Significant research efforts have been devoted to improve ALL prognostication using relevant clinical annotation from large cohorts. For example *Enshaei *et al*.* used data from four different trials involving thousands of ALL patients for the development of a continuous risk model based on white cell count at diagnosis, cytogenetics and end-of-induction MRD [47]. Despite promising results, the main limitation of this approach relies on the inclusion of post-induction MRD status, which impedes its application at the moment of diagnosis. Newer therapeutic approaches may try to optimize treatment since the beginning, which might limit the probability of developing clonal diversity as a driver of chemorrafractoriness [52]. In this regard, several previous reports have proved the usefulness of DNA methylation signatures determined at diagnosis to classify ALL patients into different molecular [26, 40] and prognostic subgroups [35, 53]. The present results indicate that DNA methylation signatures hold prognostic value in pediatric ALL regardless of the use of risk-adapted protocols that include cytogenetics, immunophenotype and MRD assessment.

A pivotal achievement of our investigation is the successful determination of the optimal cut-point for the MRP score. This critical threshold proficiently delineates a poor-prognosis group across all analyzed cohorts, underscoring the robustness and universal applicability of the MRP in risk stratification. While partially aligned with prevailing cytogenetic and molecular classifications, the MRP algorithm reconfigures risk groups with enhanced efficiency. This reclassification not only corroborates the established risk factors but also refines them, thereby presenting a more nuanced and potentially more accurate landscape of risk stratification in pediatric ALL.

The main advantage of our approach relates to the large sample size and the long-follow up of the patients. One limitation of our study, however, is the lower predictive performance of the RRP in the Nordic [40] independent validation set. The training set originated from Nordic patients treated on either the NOPHO-92 or NOPHO-2000 protocols, in which MRD measurements were not used to guide the indication of allo-HCT [6, 16]. On the contrary, MRD analysis was performed at days 29 and 79 post-induction to select candidates for allo-HCT in the NOPHO-2008 protocol [12]. Differences in treatment between the protocols may explain the lower reproducibility of the RRP, and further highlights the importance that MRD analysis plays in treatment stratification. Regardless, the prognostic value of our MRP was replicated in the cohort of patients treated on NOPHO-2008, indicating that our methylation-based MRP identifies patients who will succumb to their disease despite MRD-guided approaches. Future studies evaluating this methodology should pursue its potential enrichment with MRD data for risk stratification. Another relevant issue is the absence of a comparison between prognostic classifications based on integrative genomic profiling data. Such a comparison could be beneficial for evaluating different methods and refining the methodology further [54].

The translation of epigenetic biomarkers into clinical practice has been limited, with only a few successful examples in oncology [55]. DNA methylation is not currently performed in the clinical management for ALL, and consequently its implementation into the clinical routine will need a progressive adaptation [2]. Furthermore, optimizing epigenetic biomarkers for clinical use is not straightforward, and several factors need to be considered, such as genomic region selection, accurate DNA methylation measurements, confounding parameter identification, standardized data analysis, efficient turnaround time, and cost considerations [56]. However, the incorporation of DNA methylation signatures could offer a deeper layer of biological complexity, thereby facilitating more informed clinical decisions and potentially transforming patient care.

In conclusion, our research presents two innovative models utilizing DNA methylation data for predicting relapse and mortality risk (RRP and MRP) in pediatric ALL. These models surpass traditional cytogenetic and clinical prognostic methods in risk stratification. They also demonstrate potential synergies with diagnostic clinical data, enhancing their predictive performance. Our findings reveal that DNA methylation signatures, analyzed through ML, are reliable predictors of patient outcomes in pediatric ALL. Particularly, the MRP's capacity to extend beyond established markers exemplifies its transformative potential in clinical decision-making, suggesting more personalized and effective treatment approaches for pediatric ALL.

### Supplementary Information

**Additional file 1: Fig. S1**. a) Out-of-bag (OOB) estimations of RFS for each patient in the training set. Each line represents the RFS probability for each patient at different time points. b) Cumulative Risk Probability Score (CRPS) plots for the estimation of RFS in the training set (orange line) and stratified according to each of the relapse risk quartiles derived by the model (black lines). **Fig. S2**. Heatmaps DNA methylation beta values for each CpG site included in the relapse risk predictor (RRP). DNA methylation values of the 16 CpG dinucleotides included in the RRP in the training (a) and test (b) sets are plotted. Lateral bar plots represent the RRP score an DNAm risk group assigned to each patient. **Fig. S3**. a) Out-of-bag (OOB) estimations for OS for each patient in the training set. Each line represents the OS probability for each patient at different time points. b) CRPS plots for the estimation of OS in the training set (orange line) and stratified according to each of the mortality risk quartiles derived by the model (black lines). **Fig. S4**. Heatmap plots representing the DNA methylation beta values of each CpG included in the mortality risk predictor (MRP). DNA methylation values of the 53 CpG sites included in the MRP in the training (a) and test (b) sets are plotted. Lateral bar plots represent the MRP score and the DNAm risk group assigned to each patient. **Fig. S5**. a) Kaplan–Meier estimate for overall survival (OS) with 95% confidence interval (CI) for the independent dataset. b) Kaplan–Meier estimate for relapse-free survival (RFS) with 95% confidence interval (CI) for the independent dataset.

**Additional file 2: Table S1**. Cytogenetic classifications at the time of ALL diagnosis for the patients in the training and test sets. **Table S2**. C-indexes of the different random forest models evaluated for the prediction of RFS and OS. **Table S3**. Variable importance values for each of the CpGs in the relapse risk predictor (RRP). CpGs are listed in decreasing order of importance. **Table S4**. Patient distribution across the low- and high- relapse risk or mortality risk predictor (RRP/MRP) groups after applying cut-offs on the train, test and independent datasets. a) Low and high-RRP groups in response to relapse as outcome, b) low and high-MRP groups in response to relapse as outcome and c) low and high-MRP groups in response to death as outcome. Univariate cox regression was conducted to assess the effect of the RRP/MRP-based dichotomization on patient outcome. **Table S5**. Variable importance values for each of the CpGs in the final mortality risk predictor (MRP). Variables are depicted in decreasing order of importance. **Table S6**. Revised molecular subtype annotation analyzed by Krali et al. **Table S7**. C-indexes of the MRP in the independent dataset.

## Data Availability

DNA methylation data was retrieved from the Gene Expression Omnibus (GEO) datasets GSE49031, GSE38235 and the SciLifeLab data repository (10.17044/scilifelab.22303531). Clinical annotation for the patients is accessible through reasonable request to the original authors.

## Availability of code and materials

The source code for the models employed in this study is not publicly available due to the inclusion of sensitive information. For inquiries and access requests, please contact Jessica Nordlund (jessica.nordlund@medsci.uu.se).
