# Supplementary material for: Refining risk prediction in pediatric acute lymphoblastic leukemia through DNA methylation profiling
Source: Clin Epigenetics. 2024 Mar 28;16:49. doi: 10.1186/s13148-024-01662-6 (PMC10976833; doi:10.1186/s13148-024-01662-6)

Supplementary Figure 1. a) Out-of-bag (OOB) estimations of RFS for each patient in the training set. Each line represents the RFS probability for each patient at different time points. **b)** Cumulative Risk Probability Score (CRPS) plots for the estimation of RFS in the training set (orange line) and stratified according to each of the relapse risk quartiles derived by the model (black lines).

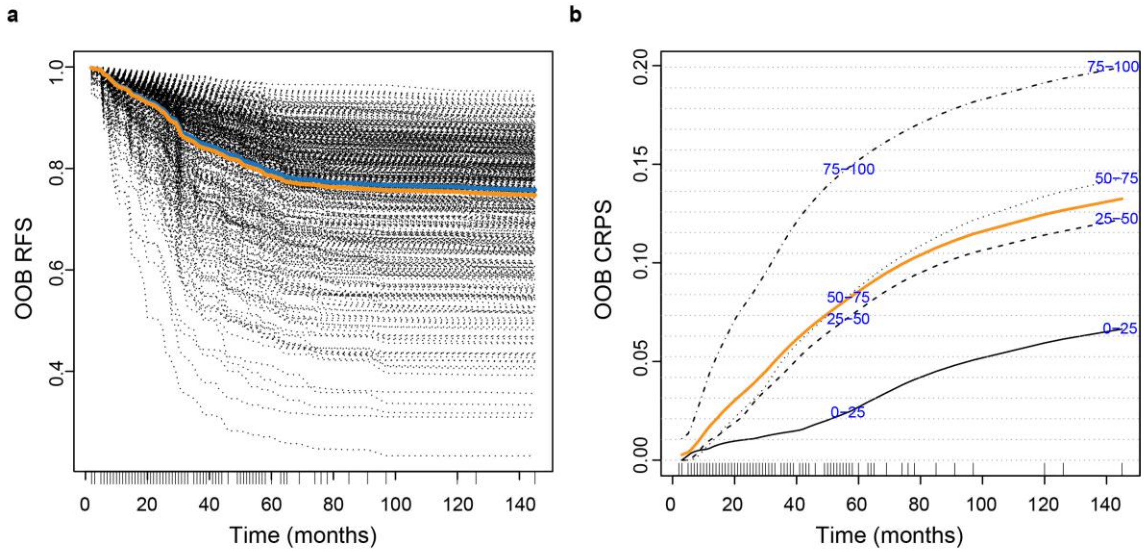

Supplementary Figure 2. Heatmaps of the DNA methylation beta values for each CpG site included in the relapse risk predictor (RRP). DNA methylation values of the 16 CpG dinucleotides included in the RRP in the training (a) and test (b) sets are plotted. Lateral bar plots represent the MRP score and the DNAm risk group assigned to each patient.

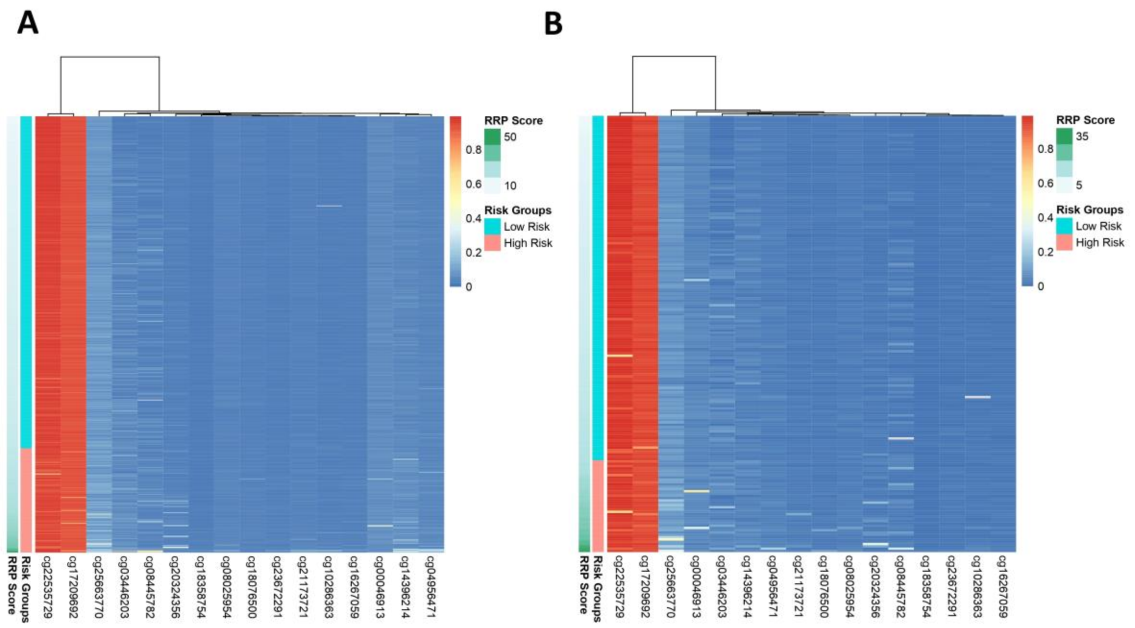

Supplementary Figure 3. a) Out-of-bag (OOB) estimations for OS for each patient in the training set. Each line represents the OS probability for each patient at different time points. b) CRPS plots for the estimation of OS in the training set (orange line) and stratified according to each of the mortality risk quartiles derived by the model (black lines).

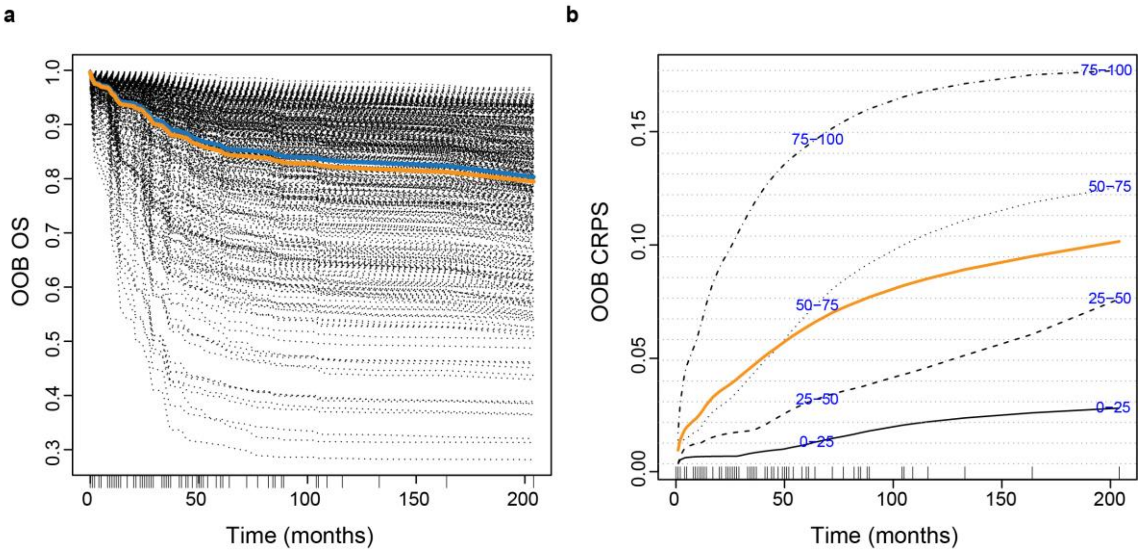

Supplementary Figure 4. Heatmap plots representing the DNA methylation beta values of each CpG included in the mortality risk predictor (MRP). DNA methylation values of the 53 CpG sites included in the MRP in the training (a) and test (b) sets are plotted. Lateral bar plots represent the MRP score and the DNAm risk group assigned to each patient.

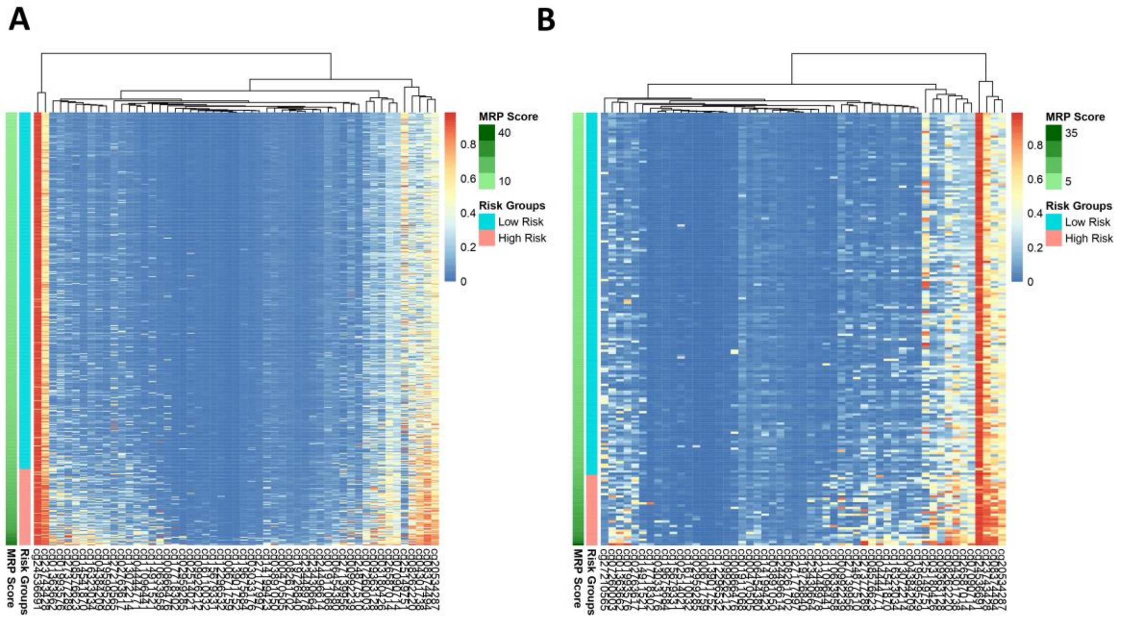

Supplementary Figure 5. a) Kaplan-Meier estimate for overall survival (OS) with 95% confidence interval (CI) for the independent dataset. b) Kaplan-Meier estimate for relapse-free survival (RFS) with 95% confidence interval (CI) for the independent dataset.

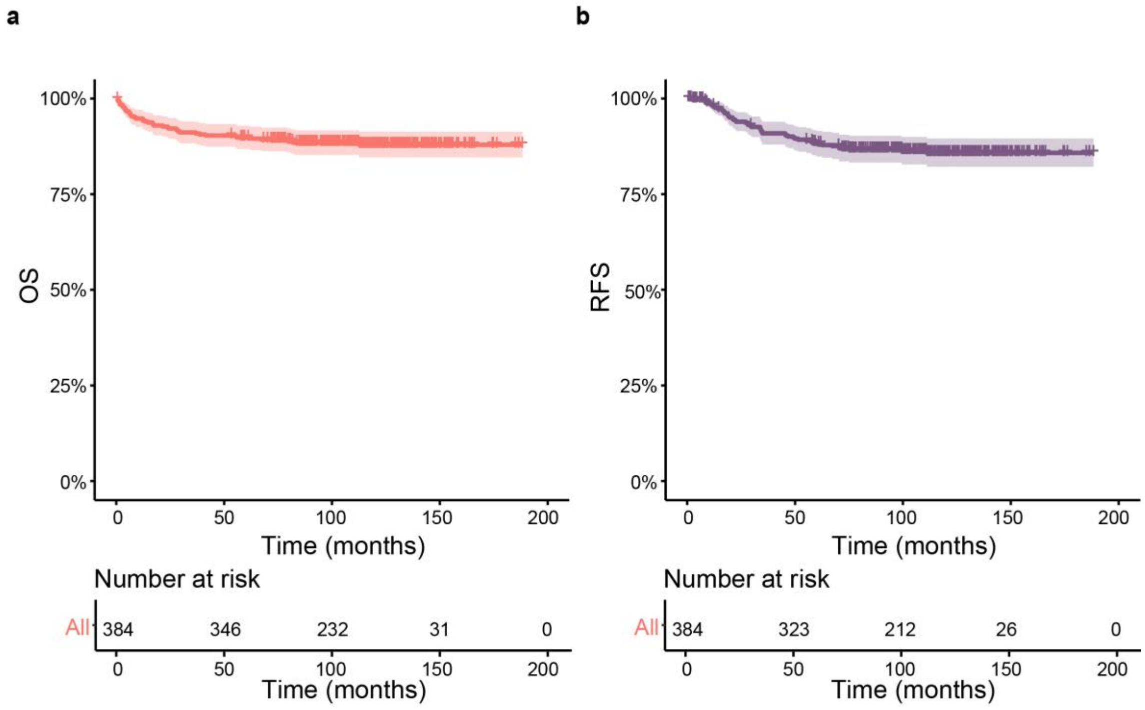

Supplement: Supplementary file 1 — Additional file 1: Fig. S1. a) Out-of-bag (OOB) estimations of RFS for each patient in the training set. Each line represents the RFS probability for each patient at different time points. b) Cumulative Risk Probability Score (CRPS) plots for the estimation of RFS in the training set (orange line) and stratified according to each of the relapse risk quartiles derived by the model (black lines). Fig. S2. Heatmaps DNA methylation beta values for each CpG site included in the relapse risk predictor (RRP). DNA methylation values of the 16 CpG dinucleotides included in the RRP in the training (a) and test (b) sets are plotted. Lateral bar plots represent the RRP score an DNAm risk group assigned to each patient. Fig. S3. a) Out-of-bag (OOB) estimations for OS for each patient in the training set. Each line represents the OS probability for each patient at different time points. b) CRPS plots for the estimation of OS in the training set (orange line) and stratified according to each of the mortality risk quartiles derived by the model (black lines). Fig. S4. Heatmap plots representing the DNA methylation beta values of each CpG included in the mortality risk predictor (MRP). DNA methylation values of the 53 CpG sites included in the MRP in the training (a) and test (b) sets are plotted. Lateral bar plots represent the MRP score and the DNAm risk group assigned to each patient. Fig. S5. a) Kaplan–Meier estimate for overall survival (OS) with 95% confidence interval (CI) for the independent dataset. b) Kaplan–Meier estimate for relapse-free survival (RFS) with 95% confidence interval (CI) for the independent dataset. [file 13148_2024_1662_MOESM1_ESM.pdf]
