# Supplementary material for: Refining risk prediction in pediatric acute lymphoblastic leukemia through DNA methylation profiling
Source: Clin Epigenetics. 2024 Mar 28;16:49. doi: 10.1186/s13148-024-01662-6 (PMC10976833; doi:10.1186/s13148-024-01662-6)
Supplement: Supplementary file 2 — Additional file 2: Table S1. Cytogenetic classifications at the time of ALL diagnosis for the patients in the training and test sets. Table S2. C-indexes of the different random forest models evaluated for the prediction of RFS and OS. Table S3. Variable importance values for each of the CpGs in the relapse risk predictor (RRP). CpGs are listed in decreasing order of importance. Table S4. Patient distribution across the low- and high- relapse risk or mortality risk predictor (RRP/MRP) groups after applying cut-offs on the train, test and independent datasets. a) Low and high-RRP groups in response to relapse as outcome, b) low and high-MRP groups in response to relapse as outcome and c) low and high-MRP groups in response to death as outcome. Univariate cox regression was conducted to assess the effect of the RRP/MRP-based dichotomization on patient outcome. Table S5. Variable importance values for each of the CpGs in the final mortality risk predictor (MRP). Variables are depicted in decreasing order of importance. Table S6. Revised molecular subtype annotation analyzed by Krali et al. Table S7. C-indexes of the MRP in the independent dataset. [file 13148_2024_1662_MOESM2_ESM.pdf]

Supplementary Table 1. Cytogenetic classifications at the time of ALL diagnosis for the patients in the training and test sets.

	Training set	Test set
B-ALL, NOS	26.7% (n = 153)	23.2% (n = 44)
B-ALL, hyperploid	24.6% (n = 141)	28.4% (n = 54)
B-ALL with t(12;21)(p13.2;q22.1)/ETV6::RUNX1	21.1% (n = 121)	22.6% (n = 43)
T-ALL	13.4% (n = 77)	12.6% (n = 24)
B-ALL with t(1;19)(q23.3;p13.3)/TCF3::PBX1	3.7% (n = 21)	0% (n = 0)
B-ALLwith t(v;11q23.3)/KMT2A rearranged	3.3% (n = 19)	4.7% (n = 9)
B-ALL, with PAX5 alteration	3.0% (n = 17)	3.2% (n = 6)
B-ALLwith t(9;22)(q34.1;q11.2)/BCR::ABL1	2.6% (n = 15)	2.1% (n = 4)
B-ALL with iAMP21	1.2% (n = 7)	2.6% (n = 5)
B-ALL, hypoploid	0.3% (n = 2)	0.5% (n = 1)

Supplementary Table 2. C-indexes of the different random forest models evaluated for the prediction of RFS and OS.

OS predictors				
**Q-value**	**HR filter**	**Correlation Filter**	**Training set c-index**	**Test set c-index**
0.05	Yes	No	0.7383	0.7374
0.05	No	No	0.7388	0.7256
0.01	Yes	No	0.7245	0.7146
0.01	No	No	0.7219	0.7189
0.05	Yes	Yes	0.7469	0.759
0.05	No	Yes	0.751	0.726
0.01	Yes	Yes	0.7082	0.6905
0.01	No	Yes	0.7141	0.7141

RFS predictors				
**Q-value**		**Correlation Filter**	**Training set c-index**	**Test set c-index**
0.05	Yes	No	0.6408	0.6856
0.05	No	No	0.6408	0.6856
0.01	Yes	No	0.6329	0.668
0.01	No	No	0.6329	0.668

Supplementary Table 3. Variable importance values for each of the CpGs in the relapse risk predictor (RRP). CpGs are listed in decreasing order of importance.

ID	Order	Variable Importance
cg25663770	1	0.06554844
cg18358754	2	0.059755976
cg20324356	3	0.055948684
cg17209692	4	0.046814949
cg22535729	5	0.046196853
cg14396214	6	0.044118723
cg03446203	7	0.041276727
cg18076500	8	0.032943851
cg21173721	9	0.028398854
cg16267059	10	0.027563885
cg08445782	11	0.02581552
cg00046913	12	0.02409957
cg08025954	13	0.022245443
cg23672291	14	0.021704871
cg04956471	15	0.018572842
cg10286363	16	0.014819689

Supplementary Table 4. Patient distribution across the low- and high- relapse risk or mortality risk predictor (RRP/MRP) groups after applying cut-offs on the train, test and independent datasets. a) Low and high-RRP groups in response to relapse as outcome, b) low and high-MRP groups in response to relapse as outcome and c) low and high-MRP groups in response to death as outcome. Univariate cox regression was conducted to assess the effect of the RRP/MRP-based dichotomization on patient outcome.

RRP cut-off on relapse data

a)	Train dataset (n = 573)						Test dataset (n = 19			
	n patients	% patients	n relapses	within group % relapses	COX regression HR*	p-value	n patients	% patients	n relapses	within group % relapses
low-RRP	436	76.1	74	17	Reference	-	150	78.9	25	16.7
high-RRP	137	23.9	55	40.1	2.982 (95% CI: 2.102-4.229)	8.98×10^{-10}	40	21.1	18	45
**Total**	573	100	129				190	100	43	

MRP cut-off on relapse data

b)	Train dataset (n = 573)						Test dataset (n = 19			
	n patients	% patients	n relapses	within group % relapses	COX regression HR*	p-value	n patients	% patients	n relapses	within group % relapses
low-MRP	472	82.4	98	20.8	Reference	-	159	83.7	31	19.5
high-MRP	101	17.6	31	30.7	2.182 (95% CI: 1.456-3.27)	0.000157	31	16.3	12	38.7
**Total**	573	100	129				190	100	43	

MRP cut-off on mortality data

c)	Train dataset (n = 573)						Test dataset (n = 19			
	n patients	% patients	n deaths	within group % deaths	COX regression HR*	p-value	n patients	% patients	n deaths	within group % deaths

**low-MRP**	472	82.4	53	11.2	Reference	-	159	83.7	15	9.4
**high-MRP**	101	17.6	44	43.6	5.25 (95% CI: 3.516-7.846)	5.51 x 10^{-16}	31	16.3	15	48.4
**Total**	573	100	97				190	100	30	

*HR: Hazard ratio

Supplementary Table 5. Variable importance values for each of the CpGs in the final mortality risk predictor (MRP). Variables are depicted in decreasing order of importance.

ID	Order	Variable Importance
cg27210565	1	0.063527541
cg24536691	2	0.042341039
cg03180426	3	0.041068496
cg02514021	4	0.040311171
cg25149751	5	0.038510061
cg14199423	6	0.034164831
cg13072214	7	0.033705229
cg00470505	8	0.032985824
cg24877510	9	0.03295808
cg04301738	10	0.028867644
cg01911068	11	0.028259842
cg01363662	12	0.027528074
cg12296532	13	0.026475847
cg11096441	14	0.02633762
cg14911521	15	0.025627303
cg27177997	16	0.024900512
cg27377289	17	0.024210814
cg03033176	18	0.023074801
cg27139956	19	0.022446827
cg08262220	20	0.021682839
cg26587014	21	0.021155954
cg04444771	22	0.019274253
cg20534287	23	0.019063137
cg17718302	24	0.018363372
cg19675684	25	0.018226099
cg09363128	26	0.018022938
cg16323034	27	0.017824248
cg24496614	28	0.017757746
cg00992239	29	0.017732871
cg10633958	30	0.017696944
cg06454380	31	0.017257257
cg23743428	32	0.016514352
cg16110032	33	0.016450912
cg08374494	34	0.015762679
cg01993576	35	0.015554267
cg00866476	36	0.015148683
cg04384209	37	0.014865501
cg19599529	38	0.014854579
cg03891050	39	0.014626172
cg19726840	40	0.014613516
cg07260003	41	0.014193535
cg00671759	42	0.013626694

cg09926212	43	0.013236141
cg02763617	44	0.012980205
cg07090714	45	0.012969178
cg08576623	46	0.012921712
cg15679331	47	0.012061588
cg23448978	48	0.012011635
cg14541870	49	0.011934702
cg23430664	50	0.011162204
cg02959285	51	0.010143257
cg13901752	52	0.009903484
cg08261702	53	0.006727811

Supplementary Table 6. Revised molecular subtype annotation analyzed by *Krali et al.*

	**NOPHO independent set**
B-ALL, hyperploid	28.1% (n = 108)
B-ALL with t(12;21)(p13.2;q22.1)/ETV6::RUNX1	26.6% (n = 102)
B-ALL, NOS	26.0% (n = 100)
B-ALLwith t(v;11q23.3)/KMT2A rearranged	7.6% (n = 29)
B-ALL with t(1;19)(q23.3;p13.3)/TCF3::PBX1	3.4% (n = 13)
B-ALL, with PAX5 alteration	2.1% (n = 8)
B-ALL, hypoploid	1.8% (n = 7)
B-ALL with iAMP21	1.8% (n = 7)
B-ALLwith t(9;22)(q34.1;q11.2)/BCR::ABL1	1.6% (n = 6)
T-ALL	1.0% (n = 4)

Supplementary Table 7. MRP c-indexes of the MRP in the independent dataset.

	Sample Size	C-index
**Standard Risk**	170	0, 590
**Intermediate Risk**	126	0, 523
**High Risk**	66	0.496
**Infant**	20	0.563
